# The gut microbiome - a new target for understanding, diagnosing and treating disease

**DOI:** 10.1186/2049-3258-72-S1-K3

**Published:** 2014-06-06

**Authors:** Jeroen Raes

**Affiliations:** 1 Department Microbiology & Immunology KU Leuven, B-3000 Leuven, Belgium

## 

The functioning of the human body constitutes a complex interplay of human processes and ‘services’ rendered to us by the 1000 trillion microbial cells we carry. Disruption of this natural microbial flora is linked to infection, autoimmune diseases and cancer, but detailed knowledge about our microbial component remains scarce [1].

Recent technological advances such as metagenomics and next-generation sequencing permit the study of the various microbiota of the human body at a previously unseen scale. These advances have allowed the initiation of the International Human Microbiome Project, aiming at genomically characterizing the totality of human-associated microorganisms (the “microbiome”) [2].

Here, I will present our work on characterizing the human intestinal flora based upon the analysis of high-throughput meta-omics (metagenomics, metatranscriptomics, metaproteomics) data. I will show how the healthy gut flora can be classified “enterotypes” that are independent from host nationality, age, BMI and gender, but linked to nutrition [3]. I will also show how metagenome-wide association studies (MGWAS) can lead to the detection of diagnostic markers for host properties and disease (e.g. in IBD, diabetes and obesity), and aid in further understanding on how the gut flora disturbances contribute to these pathologies. Finally, I will illustrate how gut microbiota-based treatment strategies are emerging, for example through Faecal Microbiota Transplantation (FMT).
